# Metabolic Adaptation in Obesity and Type II Diabetes: Myokines, Adipokines and Hepatokines

**DOI:** 10.3390/ijms18010008

**Published:** 2016-12-22

**Authors:** Kyoung-Jin Oh, Da Som Lee, Won Kon Kim, Baek Soo Han, Sang Chul Lee, Kwang-Hee Bae

**Affiliations:** 1 Metabolic Regulation Research Center, Korea Research Institute of Bioscience and Biotechnology (KRIBB), Daejeon 34141, Korea; kjoh80@kribb.re.kr (K.-J.O.); dasom89@kribb.re.kr (D.S.L.); wkkim@kribb.re.kr (W.K.K.); bshan@kribb.re.kr (B.S.H.); 2 Department of Functional Genomics, University of Science and Technology (UST), Daejeon 34141, Korea

**Keywords:** myokines, adipokines, hepatokines, obesity, type II diabetes

## Abstract

Obesity and type II diabetes are characterized by insulin resistance in peripheral tissues. A high caloric intake combined with a sedentary lifestyle is the leading cause of these conditions. Whole-body insulin resistance and its improvement are the result of the combined actions of each insulin-sensitive organ. Among the fundamental molecular mechanisms by which each organ is able to communicate and engage in cross-talk are cytokines or peptides which stem from secretory organs. Recently, it was reported that several cytokines or peptides are secreted from muscle (myokines), adipose tissue (adipokines) and liver (hepatokines) in response to certain nutrition and/or physical activity conditions. Cytokines exert autocrine, paracrine or endocrine effects for the maintenance of energy homeostasis. The present review is focused on the relationship and cross-talk amongst muscle, adipose tissue and the liver as secretory organs in metabolic diseases.

## 1. Introduction

**Figure 1:**
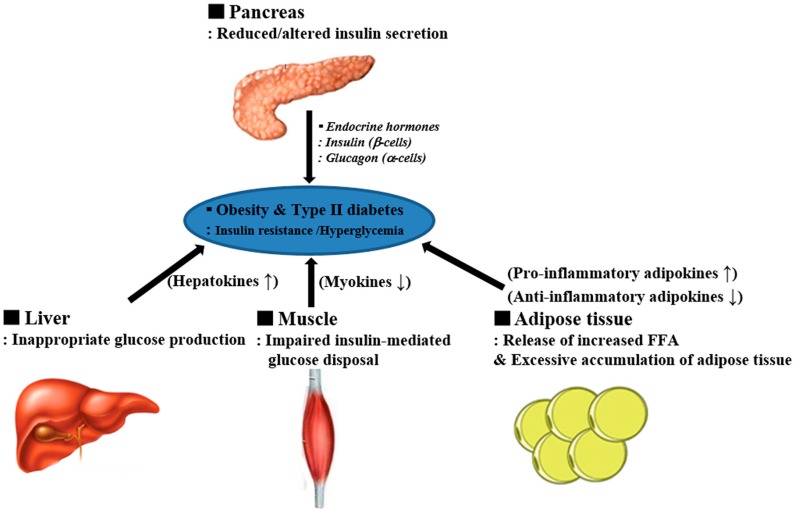
Obesity and type II diabetes are characterized by insulin resistance in peripheral tissues. Under insulin-resistant conditions, impaired insulin action enhances hepatic glucose production. Obese adipose tissue secretes adipokines that induce a pathogenic and pro-inflammatory environment. Exercise-induced myokines may prevent the harmful effects of pro-inflammatory adipokines and hepatokines. FFA: free fatty acid.

Metabolic disease is characterized by insulin resistance in peripheral tissues. Impaired insulin action increases hepatic glucose production, decreases muscle glucose uptake, and promotes lipid accumulation in insulin-sensitive organs such as muscle, liver and fat [1,2,3]. Recent evidence has identified skeletal muscle and the liver, as well as adipocytes, as secretory organs [4,5,6,7]. These metabolic organs communicate with each other regarding the regulation of energy homeostasis and insulin sensitivity. For instance, improved muscle function by exercise can affect whole-body glucose–lipid metabolism and peripheral insulin sensitivity. The manner in which contracting skeletal muscle is able to communicate with other organs and regulate directly or indirectly whole-body energy homeostasis is through myokines that are secreted from muscle and their endocrine effects. This review classifies muscle, liver and adipose tissue as endocrine organs intimately related to metabolic diseases (Figure 1). Here, we introduce myokines, adipokines and hepatokines as involved in the fundamental molecular mechanism that allows communication amongst metabolic organs in the endocrine system.

## 2. Myokines

A sedentary lifestyle (physical inactivity) increases the risk of metabolic diseases such as obesity and type II diabetes [8,9,10]. Exercise (physical activity) induces metabolic and mitochondrial adaptation to improve energy metabolism and the function of many organs [11,12]. Recently, Stanford et al. described that exercise training obviously increases energy-dissipating beige adipocytes in subcutaneous white adipose tissue (scWAT) in rodents even though its effect is controversial in human subjects [13]. Exercise-trained mice compared to sedentary mice exhibit improved whole-body glucose metabolism and mitochondrial function, indicating that exercise can induce metabolic adaptation to improve whole-body energy metabolism. These findings imply that muscle-derived factor(s) stemming from muscle contraction affect scWAT.

**Figure 2:**
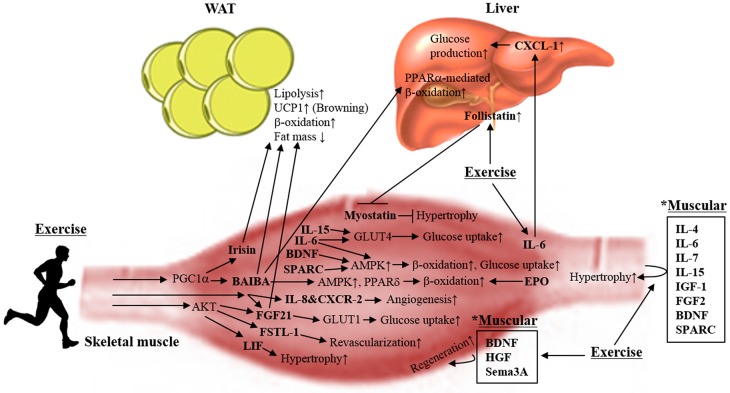
Exercise-induced myokines play critical roles in beneficial metabolic adaptations. Contracting skeletal muscles release myokines, and promote hypertrophy and myogenesis within muscle itself. The action of myokines for exercise-induced metabolic adaptation is responsible for promoting glucose uptake, glucose disposal, lipolysis, β-oxidation, angiogenesis, and revascularization. Subsequently, they exert systemic effects that improve lipid and glucose metabolism in white adipose tissue (WAT) and the liver. Specifically, muscular irisin, β-aminoisobutyric acid (BAIBA) and fibroblast growth factor 21 (FGF21) lead to the induction of “browning” in WAT that can counteract obesity and its associated metabolic diseases. UCP1: uncoupling protein 1; PGC1α: peroxisome proliferator-activated receptor gamma coactivator 1-α; FSTL-1: follistatin-related protein 1; LIF: leukemia inhibitory factor; BDNF: brain-derived neurotrophic factor; SPARC: secreted protein acidic and rich in cysteine; AMPK: AMP-activated protein kinase; PPARδ: peroxisome proliferator-activated receptor delta; GLUT1: glucose transporter 1; GLUT4: glucose transporter 4; CXCR-2: CXC chemokine receptor-2; CXCL-1: CXC chemokine ligand-1; EPO: erythropoietin; HGF: hepatocyte growth factor; Sema3A: semaphorin 3A; IGF-1: insulin-like growth factor 1; FGF2: fibroblast growth factor 2.

Skeletal muscle is one of the largest organs in the human body, comprising approximately 40% of one’s body weight. Skeletal muscle has been identified as a secretory organ [4]. Contracting muscle produces and releases a variety of cytokines and other peptides, which are collectively termed “myokines”. Myokines mediate muscle growth (myogenesis) and regeneration within the muscle itself and enable communication with other organs such as adipose tissue, liver and pancreas. Because the production of most myokines is affected by muscle contraction, physical inactivity can change the production profile of myokines and their responses. The action of myokines for exercise-induced adaptation in skeletal muscle is responsible for glucose disposal, fatty acid oxidation and lipolysis, suggesting that muscle-derived cytokines (myokines) are important for the prevention and treatment of type II diabetes (Figure 2) [11,12]. Therefore, myokines and their mechanisms can persuasively explain the negative correlation between a sedentary lifestyle and metabolic diseases such as obesity and type II diabetes.

### 2.1. Irisin

Irisin has been newly identified as a transcriptional co-activator peroxisome proliferator-activated receptor gamma coactivator 1-α (PGC1α)-dependent myokine [14]. PGC1α, a master regulator of muscle energy metabolism, enhances mitochondrial biogenesis and glucose uptake, and also plays a key role in a fiber-type switch to create oxidative fiber [15,16,17,18]. Muscle PGC1α is increased following exercise, and it regulates angiogenesis [19,20,21,22,23]. Overexpression of PGC1α in muscle stimulates the expression of the transmembrane protein fibronectin type III domain-containing 5 (FNDC5) [14]. FNDC5 is cleaved, and the cleaved ectodomain of FNDC5 is secreted from muscle. The circulating cleaved form of FNDC5 is termed irisin. Muscle-derived irisin targets some white adipocytes in white adipose tissue (WAT) and induces transdifferentiation into beige (brite) cells that express uncoupling protein 1 (UCP1) [14,24]. Therefore, irisin can cause weight loss by increasing energy expenditures. The sequential induction of PGC1α, FNDC5, and circulating irisin is increased following exercise in both humans and mice [14,24]. Recently, a FNDC treatment in obese mouse model improved glucose tolerance and induced the expression of mitochondrial genes [14]. Additionally, circulating irisin is positively correlated with muscle mass, whereas it is inversely correlated with fat mass [14]. These findings strongly suggest that irisin is a typical example of factor which provides beneficial effects through exercise-induced metabolic adaptation. On the other hand, unlike studies in rodents, the effects of irisin in humans remain controversial. Irisin can be induced but is not consistently activated by exercise in humans, and skeletal muscle FNDC5 mRNA expression is limited in response to exercise in humans [25,26]. Recently, WAT was identified as an organ that can secrete irisin, and obese patients exhibited high circulating irisin levels [27,28,29,30,31,32,33]. Furthermore, circulating irisin levels in humans were positively correlated with adiposity parameters such as body weight, fat mass, body mass index (BMI), waist-to-hip ratio and homeostasis model assessment of insulin resistance (HOMA-IR) [25,27,28,29,30,31,32,33]. These reports suggest that adiposity would be one of the main contributors to increase circulating irisin levels in humans.

### 2.2. Fibroblast Growth Factor 21

Fibroblast growth factor 21 (FGF21) was recently identified as a myokine that is produced by skeletal muscle [34,35]. Muscular FGF21 enhances glucose uptake and increases the expression of glucose transporter 1 (GLUT1) in skeletal muscle [36]. The phosphoinositide 3-kinase/protein kinase B (PI3K/AKT1) signaling pathway is linked to muscle hypertrophy [34,35]. Myogenic AKT1 induction leads to increased muscle mass, decreased fat mass, and improved whole body energy metabolism [34]. Furthermore, the expression of FGF21 was increased in the muscle and plasma of skeletal-muscle-specific AKT1 transgenic mice. After acute insulin infusion with a hyperinsulinemic euglycemic clamp, muscular and plasma FGF21 levels were increased, suggesting that insulin stimulates the expression and secretion of muscular FGF21. Recently, it was reported that exercise induces an elevated FGF21 level in plasma and muscle, and that circulating FGF21 increases lipolysis and decreases blood glucose levels [36,37,38,39]. Additionally, the expression of FGF21 was enhanced in the skeletal muscle of mice with muscle-specific deletion of *ATF7* (activating transcription factor 7), resulting in increased fatty acid oxidation, lipolysis, and the browning of WAT [40]. Muscle-specific *ATF7* knockout mouse also showed resistance against diet-induced obesity and insulin resistance owing to FGF21 induction [40]. Another example that supports FGF21 as a myokine is the elevated expression of FGF21 in skeletal-muscle-specific *UCP1* transgenic mice [41]. FGF21 induction in the muscle tissue of *UCP1* transgenic mice led to increased plasma levels of FGF21, resulting in increased browning, lipolysis and respiratory capacity in WAT [41]. These findings suggest that myokine FGF21, as a critical metabolic regulator, would be an attractive target molecule for the treatment of type II diabetes and obesity.

### 2.3. β-Aminoisobutyric Acid

β-aminoisobutyric acid (BAIBA) has been newly identified as a myokine [42]. Begriche et al. found that BAIBA improved obesity and impaired metabolic phenotypes through increased fatty acid oxidation and reduced de novo lipogenesis in a leptin-deficient *ob*/*ob* mouse model and in mice fed a high-calorie diet [43]. Recently, BAIBA, as a myokine, was found to be markedly increased in skeletal muscle and serum following exercise [42]. It was shown to mimic the beneficial effects of exercise in energy metabolism [42,43]. Naturally increased BAIBA lowered the levels of circulating blood glucose, triglycerides (TG), and cholesterol without changing the muscle structure (hypertrophy) or muscle strength [42]. It also affected metabolic changes in the liver and in adipose tissue [42,43]. BAIBA is a PGC1α-dependent myokine [42] which is released from muscle when PGC1α is expressed in muscle tissue. Exercise induces PGC1α, which is followed by the activation of peroxisome proliferator-activated receptor α (PPARα). PPARα increases fatty acid breakdown and fat utilization as an energy source in the body. This process is known as the β-oxidation signaling pathway. BAIBA improves hepatic lipid metabolism via PPARα-mediated β-oxidation and contributes to the conversion of energy-storing white adipocytes into energy-burning brown-like beige adipocytes through the activation of PPARα [42]. Furthermore, an administration of BAIBA stimulates fatty acid oxidation via AMP-activated protein kinase (AMPK) and peroxisome proliferator-activated receptor δ (PPARδ) in skeletal muscle [44], and a BAIBA treatment can restore impaired insulin signaling pathways and protect against inflammation in the skeletal muscle of mice fed a high-fat diet [44].

### 2.4. Interleukin 6

Interleukin 6 (IL-6) is the first myokine that is also known as a protein that is secreted into the blood stream during muscle contraction [45]. Skeletal muscle is the predominant source of IL-6 production [45,46,47]. During muscle contraction, the plasma concentration of IL-6 is increased by up to 100-fold, and a large amount of IL-6 is released into circulating blood [48]. Therefore, contracting skeletal muscle is the main source of circulating IL-6 in response to exercise. On the other hand, IL-6 responds well to acute exercise rather than prolonged exercise, and is increased more during eccentric exercise than during concentric exercise [49]. Muscular IL-6 promotes myogenic differentiation within skeletal muscle itself [50]. It also increases basal and insulin-stimulated glucose uptake levels by inducing the translocation of GLUT4 [46]. AMPK is an attractive therapeutic target for the treatment of obesity and type II diabetes. IL-6 promotes intramuscular and whole-body fatty acid oxidation by the activation of AMPK in both skeletal muscle and adipose tissue [51,52,53]. It also increases glucose uptake via the activation of AMPK. Further, it stimulates lipolysis in skeletal muscle but not in adipose tissue. Consistently, IL-6 knockout mouse exhibit obesity and glucose intolerance [54]. Additionally, IL-6 can function as an anti-inflammatory factor [55,56,57]. It also inhibits lipopolysaccharide (LPS)-induced tumor necrosis factor (TNF) production. Conversely, TNF levels were markedly elevated in IL-6 knockout mice and in anti-IL-6 antibody-treated mice [54,55]. These findings strongly support the contention that muscle-derived IL-6 is beneficial for the regulation of metabolic disorders such as obesity and type II diabetes. Further, muscle-derived IL-6 is associated with endogenous glucose production (hepatic glucose production) during exercise [58,59,60]. It is thought that hepatic regulation by IL-6 is mediated by another cytokine, i.e., the chemokine CXC ligand-1 (CXCL-1) [61]. It is increased in serum, muscle and liver tissues after exercise. Exercise-induced hepatic CXCL-1 was completely reduced in IL-6 knockout mice, and the overexpression of IL-6 in muscle induced CXCL-1 in the liver and serum, suggesting the presence of cross-talk between muscle and liver tissues during exercise. On the other hand, the liver is also known as a source of IL-6 production. Hepatic IL-6 increases glucose release and causes insulin resistance in the liver [60,62,63]. Adipose tissue, the brain and the connective tissue are other sources of IL-6 [64].

### 2.5. Interleukin 4

Interleukin 4 (IL-4) is a myokine which regulates myoblast fusion with myotubes and the formation and maturation of skeletal muscle [65,66]. IL-4 is expressed in muscle after strength training or by the fusion of myoblasts. Myotubes secrete IL-4, leading to myoblast fusion and growth. IL-4 is also related to muscle hypertrophy. The depletion of IL-4 in muscle cells leads to a reduction in their size and nuclear number.

### 2.6. Interleukin 7

Interleukin 7 (IL-7) is a cytokine which is involved in the development of T and B cells in the immune system. IL-7 has been identified as a myokine which is released from skeletal muscle cells [67]. It is co-expressed with the myosin heavy chain and is increased during the differentiation of human myotubes and with adaptation to strength training. IL-7 stimulates the cell migration of satellite cells and plays a critical role in myogenesis and muscle hypertrophy.

### 2.7. Interleukin 8

Interleukin 8 (IL-8) belongs to the CXC family of chemokines that possess neutrophil chemoattractant activity [68,69,70]. Muscle-derived IL-8 also stimulates angiogenesis [70,71]. It can bind to the CXC chemokine receptors CXCR-1 and CXCR-2 [68]. IL-8 exerts chemotactic activity with CXCR-1, whereas it induces angiogenesis via CXCR-2. Exercise markedly increases the expression of IL-8 and its receptor CXCR-2 within muscle itself [72]. Muscle-derived IL-8 exerts autocrine and paracrine activity, and the intramuscular expression of IL-8 is higher in contracting muscle. The plasma level of IL-8 increases in response to exhaustive exercise rather than concentric exercise.

### 2.8. Interleukin 15

Interleukin-15 (IL-15) belongs to the interleukin-2 (IL-2) superfamily. Its expression is increased in skeletal muscle and serum following strength training [73]. IL-15 is involved in skeletal muscle growth and is closely related to obesity and type II diabetes [74,75,76,77]. Physical inactivity is directly connected with the accumulation of abdominal visceral fat. IL-15 overexpression in mouse muscle reduces visceral fat mass, but not subcutaneous fat mass. It also has anabolic effects on skeletal muscle and reduces adipose tissue mass. Additionally, increased plasma levels of IL-15 in mice significantly reduces body fat mass levels without changes in the lean body mass or levels of other cytokines. These findings suggest that muscle-secreted IL-15 decreases the visceral fat mass via an endocrine system, demonstrating the muscle-fat crosstalk. Further, IL-15 increases glucose uptake in skeletal muscle and in muscle cell lines [77]. In fact, IL-15 administration increases muscle glucose uptake and glucose transporter type 4 (GLUT4) expression, suggesting that IL-15 is a critical mediator of muscle growth, hypertrophy and glucose uptake. Additionally, treadmill exercise enhances the expression of IL-15 in the skeletal muscles of obese rats fed a high-fat diet [78]. IL-15 overexpression in the skeletal muscle of diabetic zucker rats improved glucose intolerance during treadmill exercise. These data demonstrate that exercise-induced IL-15 improves glucose uptake and glucose intolerance and that IL-5 is an attractive target for the treatment of type II diabetes.

### 2.9. Myostatin

Myostatin is a myokine that is known as growth differentiation factor 8 (GDF-8). Myostatin is a highly conserved member of the transforming growth factor β (TGF-β) protein family [79]. It inhibits muscle growth and differentiation (myogenesis) in an autocrine manner [80]. Myostatin depletion leads to skeletal muscle hypertrophy and a reduction in total body fat [81,82]. Exercise diminishes myostatin expression, whereas obesity augments myostatin levels in muscle and serum [83]. In fact, myoblast isolated from obese patients secretes an excessive amount of myostatin compared to that in normal individuals [84]. Follistatin is also member of the TGF-β superfamily. It acts as an endogenous and natural inhibitor of myostatin in skeletal muscle [85]. However, contracting muscle is not a primary organ responsible for the production of follistatin. Follistatin is a hepatokine rather than a myokine. Swimming exercise in a mouse model markedly increased follistatin levels in plasma and liver tissue [6]. It was thought that the increased circulating level of follistatin may contribute to regulate myostatin levels in skeletal muscle.

### 2.10. Brain-Derived Neurotrophic Factor

Brain-derived neurotrophic factor (BDNF) is a neurotrophin that functions in neurons through Trk receptor tyrosine kinases [86]. It plays critical roles in neuronal development, synaptic plasticity, neuron growth and survival [87]. In addition, it is important for learning and memory [88,89,90,91]. Therefore, patients with Alzheimer’s disease exhibit reduced expression levels of BDNF in the hippocampus. Approximately 70% to 80% of circulating BDNF is released from the brain. It reduces food intake and lowers blood glucose levels. A low level of circulating BDNF is implicated in obesity, type II diabetes, peripheral insulin resistance and aging [92,93,94,95,96,97,98]. Recently, it has been reported that BDNF could be expressed in skeletal muscle and that it regulates muscle metabolism [95,96,97]. Exercise augments BDNF expression in skeletal muscle. However, muscle-derived BDNF is not released into the blood-stream, and instead displays autocrine/paracrine characteristics. Muscle-contraction-induced BDNF plays important roles in muscle differentiation, development, repair and regeneration. Muscular BDNF also stimulates the phosphorylation of AMPK and acetyl-CoA carboxylase β (ACCβ), and increases fat oxidation. Consequently, the activation of muscular BDNF diminishes the size of peripheral adipose tissue. Furthermore, muscular BDNF enhances glucose utilization in diabetic skeletal muscle [98]. These results indicate that BDNF, as a novel contraction-induced protein, is a potent regulator of glucose and lipid metabolism. Eventually, it will be an attractive target for improvements of metabolic diseases such as type II diabetes and obesity.

### 2.11. Leukemia Inhibitory Factor

Leukemia inhibitory factor (LIF) is a cytokine that belongs to the IL-6 superfamily. It was first identified in 1988 as a protein secreted from ascites tumor cells [99,100]. LIF is required for platelet formation, the proliferation of hematopoietic cells, and the survival and formation of neurons [101]. Recently, LIF was established as a contraction-induced myokine [102]. It was found to be expressed in skeletal muscle following exercise and to function in an autocrine or paracrine manner. Exercise-induced PI3K/mTOR/AKT1 pathway (mTOR: mammalian target of rapamycin) also regulates LIF expression in muscle [103]. LIF enhances myoblast proliferation via the induction of the transcription factors JunB and c-Myc [104,105,106]. The proliferation of muscle satellite cells plays a key role in muscle hypertrophy and regeneration. LIF regulates myogenic differentiation and muscle satellite cell proliferation via the regulation of c-Jun N-terminal kinase (JNK), Janus kinase (JAK) and signal transducer and activator of transcription 3 (STAT3) [107,108]. Further, LIF enhances myoblast survival in dystrophic muscle, suggesting that it can be an alternative therapeutic target for the treatment of skeletal muscle diseases such as muscular dystrophy [109].

### 2.12. Secreted Protein Acidic and Rich in Cysteine

Secreted protein acidic and rich in cysteine (SPARC) was initially known as “osteonectin” [110,111,112,113], as SPARC was identified in bone, and most studies of SPARC have been associated with bone. Recently, a secretome analysis of human skeletal muscle cells showed that SPARC is a secretory muscle protein [114]. SPARC is secreted into blood in conjunction with elevated protein expression levels in muscle fiber following strength training [115]. The expression of muscle-secreted SPARC was found to increase during myogenesis [114]. Interestingly, SPARC interacts with AMPK and regulates GLUT4 expression via the activation of AMPK in muscle and muscle cells [116,117]. Muscular AMPK is a critical regulator for the improvement of glucose and lipid metabolism in obesity and type II diabetes. Therefore, it is thought that muscular SPARC may regulate glucose metabolism via AMPK activation. Additionally, SPARC decreases in skeletal muscle with age, and the reduced expression of SPARC in mouse skeletal muscle is linked to muscle atrophy [118].

### 2.13. Insulin-Like Growth Factor-1 and Fibroblast Growth Factor 2

Insulin-like growth factor 1 (IGF1) and fibroblast growth factor 2 (FGF2) are well-known osteogenic factors that stimulate bone formation. Recently, it was reported that they are osteogenic myokines [119,120]. They were found to be localized in muscle tissue and myotubes, and were present in extracts from crushed muscle. IGF-1 is associated with muscle hypertrophy. FGF2 is also secreted from muscle by wounds and injuries, causing hypertrophy in adjacent muscle cells.

### 2.14. Follistatin-Related Protein 1

Follistatin-related protein 1 (FSTL-1) was described as a myokine in 2008 [121]. Myogenic AKT plays an influential role in blood vessel growth and muscle growth [122]. Muscle-specific AKT overexpression increases intramuscular and circulating serum levels of FSTL-1. Increased FSTL-1 enhances the endothelial function and revascularization via the activation of the AKT-eNOS signaling pathway. The expression and secretion of FSTL-1 are also increased in a differentiation-dependent manner in human primary skeletal muscle cells [123]. Additionally, exercise increases circulating FSTL-1 levels; and interferon γ (IFNγ) and IL-1β also stimulate FSTL-1 secretion [123].

### 2.15. Erythropoietin

Erythropoietin (EPO) was newly classified as a myokine in 2009 [124]. EPO overexpression in mouse skeletal muscle leads to increased levels in circulating blood as well as skeletal muscle. EPO overexpression in the skeletal muscle of obese mice fed a high-fat diet causes weight loss due to the reduced fat mass. These mice exhibited improved fasting insulin levels and better glucose tolerance. Further, the muscular expression of EPO increased fatty acid oxidation in skeletal muscle. Additionally, EPO was found to be produced and secreted from skeletal muscle during and following exercise, affecting neighboring muscle in a paracrine or endocrine fashion [125]. EPO, as a myokine, increases the expression of EPO receptor (EPOR) and the phosphorylation of EPOR-related JAK2, suggesting exercise-induced skeletal muscle adaptation [125]. These data describe the physiological role of EPO as a myokine for the maintenance of metabolic homeostasis.

### 2.16. Other Myokines

#### 2.16.1. Hepatocyte Growth Factor

Hepatocyte growth factor (HGF) and its receptor c-met are co-localized in activated satellite cells in regions of muscle repair [126]. HGF exists in muscle and in the muscle extracellular matrix, and it is secreted from muscle in the event of a muscle injury [127].

#### 2.16.2. Semaphorin 3A

Semaphorin 3A (Sema3A) is induced by HGF/FGF2 [128]. It is secreted from muscle satellite cells and mediates the early myogenic differentiation of satellite cell-derived myoblasts [129,130].

## 3. Adipokines

**Figure 3:**
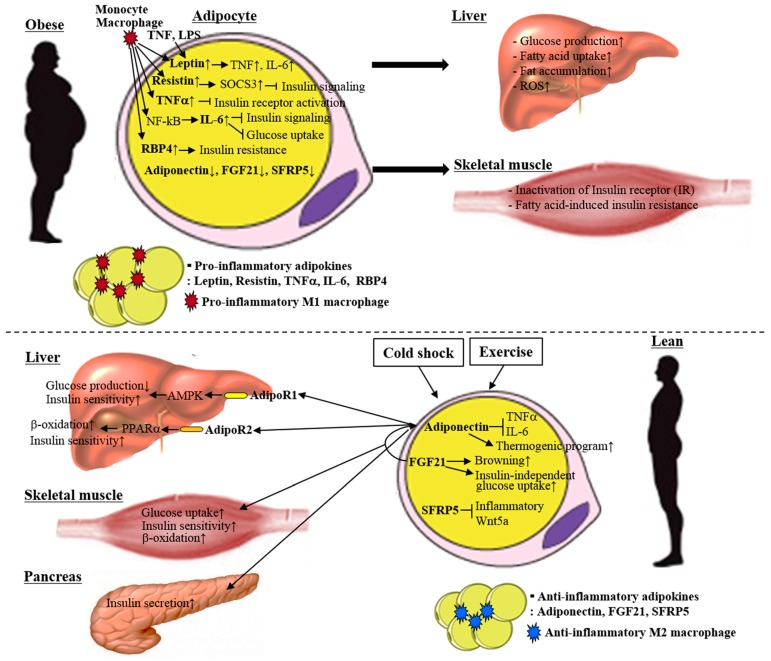
Adipokines are characterized by both pro-inflammatory and anti-inflammatory activities. Obesity increases the production of pro-inflammatory cytokines such as leptin, resistin, tumor necrosis factor α (TNFα), interleukin 6 (IL-6) and retinol binding protein 4 (RBP4) from adipose tissue. These cytokines are associated with the development of insulin resistance and metabolic abnormalities in local and peripheral tissues. On the other hand, anti-inflammatory adipokines (including adiponectin, FGF21 and SFRP5) can be stimulated by cold shock and exercise, including UCP1 expression in WAT and promoting the browning of WAT (beige). Anti-inflammatory adipokines for metabolic adaptation act systemically in an endocrine manner. They mitigate impaired lipid and glucose metabolism in the liver, muscle and pancreas, leading to improved whole-body insulin sensitivity levels. Therefore, understating the mechanism by which the balance between pro-inflammatory and anti-inflammatory adipokines can be maintained would provide a clue for researchers looking to prevent obesity and related metabolic diseases. LPS: lipopolysaccharide; SOCS3: suppressor of cytokine signaling 3; SFRP5: secreted frizzled-related protein 5; AdipoR1: adiponectin receptor 1; AdipoR2: adiponectin receptor 2.

Excess adiposity and adipocyte dysfunction are strongly associated with metabolic diseases such as obesity, type II diabetes and atherosclerosis [131,132]. WAT is an insulin-sensitive organ that stores lipids, and it produces and secretes adipocyte-specific endocrine hormones (adipokines) that can regulate energy balance levels in other peripheral tissues [133]. The main WATs are the two regional depots of visceral and subcutaneous adipose tissues, which have unique expression patterns of adipokines. Subcutaneous white adipose tissue (scWAT) is associated with an insulin sensitive phenotype, whereas visceral white adipose tissue (vWAT) is associated with obesity, type II diabetes, dyslipidemia and insulin resistance [134,135,136,137]. A sedentary lifestyle is directly linked to the accumulation of visceral fat, which is associated with the development of metabolic diseases [8,9,10]. Therefore, methods for consuming energy without physical activity have been spotlighted to promote weight loss. One of them is heat production (thermogenesis) [138,139,140]. Interestingly, there are energy-consuming white adipocytes that exhibit UCP1-dependent thermogenic capacity, similar to brown adipocytes [139,140]. These are beige adipocytes, UCP1-expressing adipocytes in scWAT. Beige adipocytes have been considered as attractive therapeutic targets for the treatment of obesity and type II diabetes, as they are found in WAT, which stores lipids. In this review, we categorize adipokines into the anti-inflammatory and inflammatory types, and describe the roles of adipokines in diet-induced metabolic changes and exercise-induced metabolic adaptation (Figure 3). We also introduce adiponectin and FGF21 as adipokines that lead to the activation of beige cells.

### 3.1. Anti-Inflammatory Adipokines

#### 3.1.1. Adiponectin

Adiponectin is one of the well-characterized classic adipokines. The gene that codes for adiponectin is located on chromosome 3q27, which is associated with type II diabetes and metabolic syndrome [141,142]. There are at least three homomeric complexes in blood: trimer (LMW: the low-molecular-weight form), hexamer (MMW: the medium-molecular-weight form) and multimer (HMW: the high-molecular-weight form) [143,144]. Among them, the stable HMW form is well-known as a biologically active form for the regulation of glucose homeostasis, insulin sensitivity and metabolic homeostasis [145,146]. Adiponectin expression by adipocytes is decreased in obese individuals and is inhibited by pro-inflammatory cytokines such as TNF and IL-6 and by conditions such as hypoxia and oxidative stress [147,148,149,150,151,152]. The plasma levels of adiponectin were found to be reduced in individuals with obesity, type II diabetes and insulin resistance [153,154,155], and they are also inversely correlated with BMI [156]. In short, increased levels are associated with weight loss, whereas decreased levels are linked to weight gain [157,158]. Chronic cold exposure promotes the production of anti-obese hormone adiponectin in scWAT. Cold-induced adiponectin regulates the thermogenic program through the proliferation of M2 macrophages, leading to the browning of scWAT [159]. Further, adiponectin enhances glucose uptake and fatty acid oxidation in skeletal muscle and suppresses glucose production in the liver via the activation of AMPK [160,161]. Additionally, adiponectin stimulates insulin secretion in vivo, and hypoadiponectinemia causes β-cell dysfunction [162,163,164]. In line with these findings, mice lacking adiponectin exhibit hepatic insulin resistance and exacerbated diet-induced resistance. Adiponectin exerts beneficial effects through the activation of its two receptors, adiponectin receptor 1 (AdipoR1) and adiponectin receptor 2 (AdipoR2) [165,166]. AdipoR1 is expressed in several tissues and is associated with the activation of AMPK, resulting in reduced glucose production and improved insulin resistance. AdipoR2 is mainly expressed in the liver. It is related to the activation of PPARα, which is related to increased fatty acid oxidation and improved insulin sensitivity. Both receptors are related to insulin sensitivity and glucose/lipid metabolism. On the other hand, exercise has controversial effects on adiponectin and plasma adiponectin levels. It was reported that plasma adiponectin levels in obese men were significantly increased by acute aerobic exercise training [167]. In contrast, other reports found that mild or moderate exercise did not affect adiponectin levels, and high-intensity exercise decreased total adiponectin concentrations by reducing LMW and MMW adiponectin levels, but not HMW levels [168,169,170]. These data suggest that the intensity and duration of exercise are important for the regulation of adiponectin levels.

#### 3.1.2. Fibroblast Growth Factor 21

FGF21 was recently introduced as a novel adipokine [171,172,173]. Cold exposure induces FGF21 expression in WAT and brown adipose tissue (BAT), resulting in the thermogenic recruitment of WAT [174,175,176,177]. Adipose-derived FGF21 in an autocrine/paracrine manner increases the expression of uncoupling protein 1 (UCP1) and other thermogenic genes in WAT. Beige adipocytes are UCP1 positive cells in scWAT following cold exposure. FGF21 knockout mice showed a reduced level of beige adipocytes. Similarly, FGF21 induced increased energy expenditure in BAT, and FGF21 stimulated insulin-independent glucose uptake in peripheral tissues. In humans, cold exposure increases circulating FGF21 levels, resulting in the activation of brown adipocytes and lipolysis and the browning of WAT. It has also been reported that adiponectin secreted from adipocytes regulates glucose and lipid metabolism in the liver and muscle in an endocrine fashion [178,179,180,181], while adipose-derived FGF21 stimulates adiponectin expression in WAT and the blood stream [182,183]. However, the beneficial effects of FGF21 on obesity-induced insulin resistance in liver and muscle were not exhibited in adiponectin knockout mouse, suggesting that adiponectin is a downstream effector molecule of FGF21.

#### 3.1.3. Secreted Frizzled-Related Protein 5

Secreted frizzled-related protein 5 (SFRP5) is newly identified as an adipokine with anti-inflammatory effects [184]. SFRP5 is highly expressed in WAT compared to other tissues. It has been reported that the canonical Wnt family suppresses adipogenesis, while non-canonical Wnt5a enhances the inflammatory signaling pathway [185,186,187,188]. SFRP5 expression was decreased, whereas Wnt5a expression was increased in the WAT of obese mice and humans [189]. SFRP5 binds to and inhibits Wnt5a as induced by obesity. SFRP5-depleted mice exhibit insulin resistance and a fatty liver even when they are normoglycemic. Further, SFRP5 depletion under a high-fat and high-sucrose feeding condition enhances macrophage accumulation and the expression of pro-inflammatory cytokines though the Wnt5a-induced activation of inflammatory JNK1. These data suggest that SFRP5 is a beneficial target for mitigating obesity-induced adipose tissue inflammation and metabolic disorder.

### 3.2. Inflammatory Adipokines

#### 3.2.1. Leptin

Leptin is a classic adipokine which is predominantly secreted from adipocytes into the bloodstream. It is a pro-inflammatory adipokine that has been identified in *ob*/*ob* mice (leptin deficient mice) [190,191]. Leptin-deficient *ob*/*ob* mice exhibit increased food intake, decreased energy expenditure, dyslipidemia, obesity and insulin resistance. In line with these findings, leptin administration has been shown to improve lipodystrophy and insulin resistance [192,193]. However, there was a positive correlation between leptin levels in blood and adipose masses, and obese individuals have high leptin levels in the blood [194]. Thus, leptin is a well-known marker for obesity. Further, leptin enhances the production of TNF and IL-6 by monocytes [195]. It also promotes the production of reactive oxygen species (ROS) and stimulates cell proliferation and migratory responses in monocytes. In macrophages, leptin stimulates the production of CC-chemokine ligands by activating the JAK2/STAT3 signaling pathway [196]. Leptin levels in adipose tissue and plasma were increased by pro-inflammatory stimuli such as TNF and lipopolysaccharide (LPS) [197,198,199,200]. Chronic inflammation and increased TNFα play an important role in hyperleptinemia in obese individuals with leptin resistance. Several reports have described that leptin mRNA levels in adipose tissue following exercise did not change [201,202,203,204]. However, plasma leptin levels tend to decrease following exercise in patients with type II diabetes [205,206].

#### 3.2.2. Resistin

Resistin is an adipokine that functions as a pro-inflammatory biomarker and a mediator of obesity-related insulin resistance [207]. It is positively correlated with fat mass and causes endothelial dysfunction by enhancing oxidative stress. It has two quaternary forms, i.e., a less abundant trimer and an abundant hexamer [208]. The trimer form is more bioactive and is strongly associated with the induction of hepatic insulin resistance. Resistin-deficient mice exhibit low glucose levels due to the reduction of hepatic glucose production [209]. Resistin-deficient *ob*/*ob* mice show improved glucose tolerance and insulin sensitivity, although they showed increased obesity as well [210]. Resistin in the regulation of glucose metabolism is associated with the activation of a suppressor of cytokine signaling 3 (SOCS3), an inhibitor of insulin signaling [211]. Resistin levels in adipocytes and blood are not affected by exercise [212,213]. However, regular weight training reduces plasma resistin levels. Resistin promotes insulin resistance in mice, whereas whether it does so in humans is unclear [214,215,216,217]. Resistin protein synthesis in mice is restricted to adipocytes, while in humans it is generated by macrophages and monocytes, but not adipocytes. Therefore, it is thought that the role of resistin is very different in mice and humans.

#### 3.2.3. Tumor Necrosis Factor α

TNFα is an inflammatory cytokine that is produced by monocytes and macrophages. In obese individuals, macrophage-infiltrated visceral fat is the main site for TNFα production [218,219]. Visceral fat obesity is associated with a reduction of insulin sensitivity and anti-inflammatory cytokines. TNF expression is increased in the adipose tissue of humans and in a mouse model with obesity and type II diabetes [220,221]. TNFα depletion in *ob*/*ob* genetic or diet-induced obese mice reduces insulin resistance and improves insulin signaling in adipose tissue and muscle [221]. TNF attenuates the insulin-stimulated tyrosine phosphorylation of insulin receptor (IR) and insulin receptor substrate 1 (IRS1) in adipose tissue and muscle, resulting in the occurrence of insulin resistance. Patients with diabetes had elevated levels of TNFα in plasma and muscle [222,223,224,225,226]. Increased TNFα levels stimulate hepatic fatty acid uptake and increase fat accumulation and ROS production in the liver. Additionally, TNFα enhances the incorporation of fatty acids into diacylglycerol (DAG), suggesting TNFα-induced insulin resistance in skeletal muscle [227]. Exercise reduces circulating plasma levels of TNFα, whereas it does not affect TNFα expression in adipose tissue [228,229,230].

#### 3.2.4. Interleukin 6

IL-6 has both pro-inflammatory effects as an adipokine and anti-inflammatory effects as a myokine [231,232,233,234]. The reason IL-6 can have different functions in different organs would be that induction of IL-6 expression is stimulated by the different inducers and inducing signals in different organs. As mentioned in the myokine section of this review, an increase in the plasma IL-6 level following exercise mainly results from it being secreted from skeletal muscle. Muscle-derived IL-6 improves glucose and lipid metabolism and the insulin signaling pathway. On the other hand, circulating plasma IL-6 levels are elevated in individuals with type II diabetes, obesity and insulin resistance [235,236]. IL-6 as an adipokine is positively related to BMI [237]. It has been reported that one third of the circulating IL-6 level was derived from adipose tissue. Specifically, visceral adipose tissue is an important source of circulating IL-6 in relation to obesity [237,238,239]. The expression of IL-6 in adipose tissue is produced by macrophages and is stimulated by the activation of the nuclear factor κ-light-chain-enhancer of activated B cells (NF-κB) signaling pathway. Further, IL-6 impedes insulin signaling and reduces insulin-dependent glucose uptake by the inhibition of GLUT4 and IRS1 expression in adipocytes. Finally, adipocyte-derived IL-6 is related to the occurrence of insulin resistance and metabolic disorder.

#### 3.2.5. Retinol Binding Protein 4

Retinol binding protein 4 (RBP4) is a hepatocyte-secreted factor [240,241,242]. It delivers retinol from the liver to peripheral tissues as a carrier for the transport of retinol (vitamin A alcohol) [242]. Recently, RBP4 was described as an adipokine which is secreted from adipocytes [243]. It is also secreted by macrophages [244]. Adipocyte-derived RBP4 impedes the insulin-induced phosphorylation of IRS1 in an autocrine or paracrine fashion [243]. Further, adipocyte-specific GLUT4 knockout mice exhibit increased adipose expression levels of RBP4 [243]. Increased adipose RBP4 contributes to glucose intolerance and insulin resistance in adipocyte-specific GLUT4 knockout mice. The circulating serum RBP4 level is increased under insulin-resistant conditions. Under insulin resistant conditions, the serum RBP4 level is mainly produced by visceral adipose tissue and is associated with an increased BMI [244,245,246,247,248]. An increased serum level of RBP4 elevates blood pressure and plasma levels of cholesterol and TG. Therefore, RBP4 is considered as a marker of intra-abdominal fat accumulation and obesity-induced inflammation.

### 3.3. Other Adipokines

#### 3.3.1. Irisin

Adipose tissue, as well as skeletal muscle, is also the main site that can generate and secrete FNDC5/irisin [249,250,251,252]. Irisin secretion from scWAT and vWAT was induced by short-term periods of endurance exercise training, whereas it was decreased by long-term exercise training and fasting [250,251]. Obese animals such as zucker rat and diet-induced obese animal models excessively secreted irisin from WAT [250]. In humans, irisin levels were higher in obese patients compared with normal weight subjects, and body fat mass was positively correlated with circulating irisin levels [252]. These suggest that irisin secreted from WAT might play a role in metabolic pathology related with obesity and insulin resistance.

#### 3.3.2. α2-HS-Glycoprotein (Fetuin-A)

Fetuin-A, one of the liver secretory glycoproteins, stimulates the production of inflammatory cytokines from adipocytes and macrophages [253,254]. Recently, it has been reported that free fatty acid (FFA) enhanced fetuin-A expression, and elevated fetuin-A functioned as an endogenous ligand of Toll-like receptor 4 (TLR4) [255,256,257]. Further, FFA-induced fetuin-A activated TLR4-mediated inflammatory and insulin resistance pathways in adipose tissue [257]. Additionally, FFA-treated adipocytes released fetuin-A into the medium, and lipid-induced fetuin-A increased macrophage migration into adipocyte and adipose tissue [258]. Consistently, expression and secretion of fetuin-A were increased in obese and pro-inflammatory adipose tissues [257,258,259]. It has also been reported that visceral fat expressed and secreted more fetuin-A than subcutaneous fat, and excess secretion of fetuin-A inhibited insulin signaling [260]. These data indicate that fetuin-A is an inflammatory adipokine.

## 4. Hepatokines

**Figure 4:**
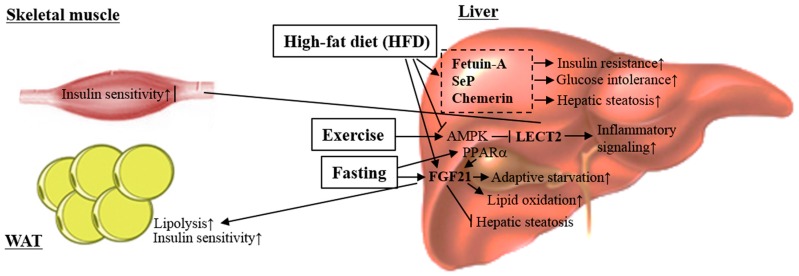
Hepatokines are closely related to the development of abnormal glucose and lipid metabolism in those with obesity. Most hepatokines, including fetuin-A, selenoprotein P (SeP), chemerin and leukocyte cell-derived chemotaxin 2 (LECT2), increase hepatic fat accumulation and activate inflammatory signaling in the liver. Further, they exacerbate hepatic glucose metabolism and insulin signaling. On the other hand, FGF21, as a hepatokine, promotes lipid oxidation, inhibits hepatic steatosis, and improves obesity-induced insulin resistance. Moreover, hepatic FGF21 stimulates lipolysis and improves obesity-induced insulin resistance in WAT.

Liver is the main insulin-sensitive organ to be in charge of glucose production [261]. Under insulin-resistant conditions, impaired insulin action promotes hepatic glucose production and decreases glucose uptake in peripheral tissues such as muscle and fat, resulting in hyperglycemia (high blood glucose) [262,263], i.e., type II diabetes. Impaired insulin action promotes excessive fat accumulation [264], that is to say, obesity. Recently, it was reported that hepatokine, a secretory protein released from the liver, could affect muscle and fat metabolic phenotypes in an endocrine-dependent manner (Figure 4) [5]. In this section, we introduce the role of hepatokines in metabolic regulation under insulin-resistant conditions.

### 4.1. α2-HS-Glycoprotein (Fetuin-A)

Fetuin-A is the first liver-derived protein (hepatokine) to show relationship with metabolic diseases. It is mainly synthesized in the liver and secreted into the bloodstream [265,266,267]. It is a phosphorylated glycoprotein that is also a natural inhibitor of insulin receptor tyrosine kinase, leading to insulin resistance [268,269,270,271,272]. Fetuin-A was positively linked to liver fat accumulation and negatively related to insulin sensitivity [273,274]. Fetuin-A levels are higher in patients with non-alcoholic fatty liver disease (NAFLD) and type II diabetes [275,276,277]. Depletion of fetuin-A improves the insulin signaling pathway in the liver and in skeletal muscle [272]. Fetuin-knockout mice exhibit improved glucose and insulin tolerance and are resistant to high-fat diet-induced weight gain. In hepatocytes, palmitate enhances NF-κB recruitment to the fetuin-A promoter, resulting in increased fetuin-A synthesis and secretion levels [274]. Circulating fetuin-A is also related to hepatic steatosis, impaired glucose tolerance, and insulin resistance [276,277].

### 4.2. Fibroblast Growth Factor 21

As above mentioned, FGF21 plays roles as a myokine and an adipokine. FGF21 also functions as a hepatokine. FGF21 is predominantly synthesized and expressed in the liver and is secreted into the bloodstream [278]. FGF21, as a fasting-induced hormone, is necessary for adaptive starvation [279]. Hepatic and circulating levels of FGF21 are increased by fasting and a high-fat/low-carbohydrate ketogenic diet [279,280]. FGF21 alleviates endoplasmic reticulum stress or obesity-induced hepatic steatosis [281,282,283,284]. The hepatic knockdown of FGF21 leads to a fatty liver and dyslipidemia, whereas the hepatic expression of FGF21 enhances hepatic lipid oxidation and TG clearance [284]. The hepatic expression of FGF21 can be stimulated by PPARα [285]. Hepatic FGF21, as an endocrine hormone, triggers lipolysis in white adipose tissue, functioning in the brain to reduce physical activity [285].

### 4.3. Leukocyte Cell-Derived Chemotaxin 2

Leukocyte cell-derived chemotaxin 2 (LECT2) is a factor that is related to hepatic inflammatory signaling and the homeostasis of hepatic natural killer T cells [286,287]. Recently, a DNA chip analysis from liver biopsies of patients with type II diabetes showed a positive correlation between the hepatic LECT2 expression level and BMI, describing a relationship between LECT2 and obesity [288]. Further, high-fat diet feeding increased serum and hepatic LECT2 levels by impeding AMPK activation [288]. On the other hand, exercise reduced hepatic and circulating levels of LECT2 via AMPK activation. LECT2 functioned as a hepatokine connected to obesity through the induction of insulin resistance in skeletal muscle.

### 4.4. Selenoprotein P

Selenoprotein P (SeP) is an abundant extracellular glycoprotein [289,290]. It is mainly produced in the liver, and it is released into plasma. A DNA chip analysis of liver biopsies from patients with type II diabetes showed that hepatic SeP expression is linked to insulin resistance [291]. Administration of SeP to mice impeded insulin signaling, and inhibited AMPK activation in the liver [292]. In contrast, depletion of SeP in mice improved insulin sensitivity and glucose tolerance. Further, a palmitate treatment increased SeP expression in hepatocytes, and the AMPK-mediated phosphorylation of FoxO1a inhibited palmitate-induced SeP expression and insulin resistance. Further, circulating SeP levels were increased in patients with NAFLD, type II diabetes and visceral obesity [293,294].

### 4.5. Chemerin

Chemerin is known as an adipokine associated with obesity [295]. Circulating levels of chemerin are increased in those with NAFLD [296]. Recently, it was reported that chemerin is produced in the liver and contributes to impaired glucose homeostasis [297]. Paigen diet feeding in conjunction with non-alcoholic fatty liver disease due to higher cholesterol contents promotes the hepatic expression of chemerin [298]. Methionine choline-deficient (MCD) diet feeding to induce a severe fatty liver due to a lack of methionine and choline increases chemerin expression levels in the liver [298]. Further, the hepatic expression of chemerin is elevated in patients with NAFLD and non-alcoholic steatohepatitis (NASH) [299]. These findings support the contention that chemerin as a hepatokine is associated with the development of hepatic steatosis.

## 5. Concluding Remarks

Understanding the regulation of endocrine hormones secreted from each metabolic organ is important for improving metabolic diseases such as obesity and type II diabetes, as metabolic diseases are not a local problem. Metabolic organs secrete cytokines in response to nutrition and physical activity, and communicate with each other for the maintenance of energy homeostasis. In this review, we demonstrate that a sedentary lifestyle combined with excessive calorie intake alters the pattern of cytokine secretion from many organs. As mentioned above, myokines, muscle-derived factors due to muscle contraction, are negatively correlated with a sedentary lifestyle and metabolic disease and are in charge of exercise-induced adaptation. They stimulate glucose disposal, fatty acid oxidation and lipolysis in other metabolic organs as well as in muscle. These reports show the whole-body adaptive effects and the health-promoting effects of exercise-induced myokines. There were a few in-depth studies to identify novel myokines and to determine their physiological functions [300,301,302]. Secretome analysis using DNA microarray and proteomics approaches identified novel exercise-induced myokines such as CX3CL1 and CCL2 [300,301]. As a lipid vesicle delivery system for exercise-induced molecules, exosomes and/or microvesicles containing exerkines (exercise-induced peptides and nucleic acids, lipids and microRNA species) facilitate the exchange of these exerkines between cells and tissues, and enable exerkines to exert systemic beneficial effects in a stable condition [302]. Indeed, secretome analyses for identifying novel myokines and a newly devised delivery system would provide an effective method for the improvement and treatment of metabolic disease. As mentioned above, myokines such as irisin and IL-6 are additionally known to be secreted by adipocytes [249,303,304]. We termed these proteins adipomyokines. They function as both pro-inflammatory mediators (adipokines) under obese conditions and beneficial effectors (myokines) following exercise. There were efforts to identify adipomyokines by using expression profiling and human secretome data [304]. Candidates for adipomyokines were biological molecules that are related with extracellular matrix (ECM) remodeling and tissue fibrosis. It has been previously reported that excessive ECM deposition in adipose tissues, adipose tissue dysfunction and fibrosis are closely associated with development of obesity-associated insulin resistance and type II diabetes [305,306,307]. Understanding adipose-muscle crosstalk in obesity and type II diabetes, and in exercise-induced metabolic adaptation would provide a more comprehensive view for the maintenance of metabolic homeostasis. Adipokines, adipocyte-derived factors, have both anti-inflammatory and pro-inflammatory effects. An imbalance between the two types of adipokines causes the abnormal expansion of adipose tissue, the leading cause of obesity, and causes local and systemic inflammation. On the other hand, there are two regional depots of adipose tissue. One is scWAT, which is involved in insulin sensitivity. Exercise can increase the production of energy-consuming beige adipocytes in scWAT. The other one is vWAT, which is closely connected to the development of insulin resistance, obesity and type II diabetes. Comparative secretome analysis proved the existence of location-specific secreted proteins in different adipose spots, scWAT and vWAT, by genome-wide mRNA expression profiling and quantitative proteomic approach. These studies support depot-specific metabolic function and distinctive features of scWAT and vWAT [308,309,310,311,312]. Recently, hepatokines, secretory proteins produced in the liver, redefined the liver as an endocrine organ that can affect and communicate with many other organs. Hepatokines play critical roles in hepatic fat accumulation and insulin resistance. However, there remains a lack of research on hepatokines.

This review will provide a better understanding of molecular mechanisms and the progression of metabolic diseases, although it described the roles of myokines, adipokines and hepatokines from skeletal muscle, adipocytes and the liver, respectively, only in a limited manner.
